# Breastfeeding and Its Prospective Association with Components of the GH-IGF-Axis, Insulin Resistance and Body Adiposity Measures in Young Adulthood – Insights from Linear and Quantile Regression Analysis

**DOI:** 10.1371/journal.pone.0079436

**Published:** 2013-11-13

**Authors:** Anke L. B. Günther, Helena Walz, Anja Kroke, Stefan A. Wudy, Christina Riedel, Rüdiger von Kries, Gesa Joslowski, Thomas Remer, Guo Cheng, Anette E. Buyken

**Affiliations:** 1 Fulda University of Applied Sciences, Department of Nutritional, Food and Consumer Sciences, Fulda, Germany; 2 Justus-Liebig-University of Giessen, Center of Child and Adolescent Medicine, Laboratory for Translational Hormone Analytics in Pediatric Endocrinology, Peptide Hormone Research Unit, Giessen, Germany; 3 Ludwig-Maximilians-University Munich, Institute of Social Paediatrics and Adolescent Medicine, Munich, Germany; 4 University of Bonn, IEL-Nutritional Epidemiology, DONALD Study at the Research Institute of Child Nutrition, Bonn, Germany; 5 West China School of Public Health, Sichuan University, Chengdu, People's Republic of China; John Hopkins Bloomberg School of Public Health, United States of America

## Abstract

**Background:**

Breastfeeding may lower chronic disease risk by long-term effects on hormonal status and adiposity, but the relations remain uncertain.

**Objective:**

To prospectively investigate the association of breastfeeding with the growth hormone- (GH) insulin-like growth factor- (IGF) axis, insulin sensitivity, body composition and body fat distribution in younger adulthood (18–37 years).

**Design:**

Data from 233 (54% female) participants of a German cohort, the Dortmund Nutritional and Anthropometric Longitudinally Designed (DONALD) Study, with prospective data on infant feeding were analyzed. Multivariable linear as well as quantile regression were performed with full breastfeeding (not: ≤2, short: 3–17, long: >17 weeks) as exposure and adult IGF-I, IGF binding proteins (IGFBP) -1, -2, -3, homeostasis model assessment of insulin resistance (HOMA-IR), fat mass index, fat-free mass index, and waist circumference as outcomes.

**Results:**

After adjustment for early life and socio-economic factors, women who had been breastfed longer displayed higher adult IGFBP-2 (p_trend_ = 0.02) and lower values of HOMA-IR (p_trend_ = 0.004). Furthermore, in women breastfeeding duration was associated with a lower mean fat mass index (p_trend_ = 0.01), fat-free mass index (p_trend_ = 0.02) and waist circumference (p_trend_ = 0.004) in young adulthood. However, there was no relation to IGF-I, IGFBP-1 and IGFBP-3 (all p_trend_>0.05). Associations for IGFBP-2 and fat mass index were more pronounced at higher, for waist circumference at very low or high percentiles of the distribution. In men, there was no consistent relation of breastfeeding with any outcome.

**Conclusions:**

Our data suggest that breastfeeding may have long-term, favorable effects on extremes of adiposity and insulin metabolism in women, but not in men. In both sexes, breastfeeding does not seem to induce programming of the GH-IGF-axis.

## Background

Whether breastfeeding plays a causal role in the prevention of overweight is still matter of scientific debate [1]. Several meta-analyses of observational studies have concluded that breastfeeding could have a small protective effect on later overweight risk [2]–[5], but evidence is not convincing that this persists until adulthood [6]. Randomized controlled trials would represent the gold-standard to address these issues, since they can overcome the drawback of residual confounding in observational studies [7]. However, they are not ethical in the case of breastfeeding [8]. One exception is randomization to breastfeeding promotion, as was done in the Belarusian PROBIT Trial. In this trial, prolonged breastfeeding did not affect adiposity measures, but the trial has not yet followed participants until adulthood and lacks statistical power [1], [9], [10].

One proposed mechanism linking breastfeeding to obesity development is a “programming” of insulin metabolism and the growth hormone- (GH) insulin-like growth factor- (IGF) axis. The GH-IGF-axis, particularly IGF-I and the binding proteins modulating its acute and long-term bioavailability, plays a central role in the regulation of human growth and glucose metabolism [11]. Breastfed infants display lower values of both insulin and IGF-I [12]–[15]. While this may favorably influence their body composition [16], it could also yield long-term metabolic adaptations, resulting in differences still discernible in adulthood. Such a programming effect of breastfeeding on the GH-IGF-axis and/or insulin metabolism could also represent an intermediary factor linking infant feeding to chronic diseases such as cancer and cardiovascular disease [17], [18], [16]. However, evidence on the relevance of breastfeeding for components of the GH-IGF-axis or insulin resistance in adulthood is sparse [19].

Regarding the long-term health effects of breastfeeding, alternative statistical approaches for analysis of continuous outcomes may provide novel insights. Using the procedure of quantile regression, Beyerlein and colleagues [20] showed that breastfeeding may exert regression-to-the-mean effects by differentially affecting the lower and upper parts of the BMI distribution in childhood. Since the association of IGF-I with total mortality risk has been reported to be U-shaped (i.e. differential across the IGF-I distribution) [21], quantile regression may also provide additional insights when addressing the relevance of breastfeeding for the adult GH-IGF-axis.

Therefore, using traditional linear and as well as quantile regression models our aim was to prospectively investigate the associations between breastfeeding and components of the GH-IGF-axis: IGF-I (a main regulator of growth in early life by mediating the effects of GH), IGFBP-1 and IGFBP-3 (important regulators of acute and longer-term IGF-I bioavailability, respectively) and IGFBP-2 (modulating IGF-1 action and reflecting long-term insulin sensitivity [11]). In addition, we considered associations of breastfeeding with insulin resistance (homeostasis model assessment for insulin resistance, HOMA-IR), body composition (fat mass index, FMI; fat-free mass index, FFMI), and body fat distribution (waist circumference, WC). Data came from the Dortmund Nutritional and Anthropometric Longitudinally Designed (DONALD) Study.

## Methods

### Ethics statement

The DONALD Study was approved by the Ethics Committee of the University of Bonn. Written consent was given by parents or adult participants, respectively, for the examinations to be performed and for their information to be stored and used for research.

### Study population

The DONALD Study is an ongoing, open cohort study conducted in Dortmund, Germany. Details on this study have been described elsewhere [22], [23]. Every year, an average of 35-40 infants are newly recruited and first examined at the age of 3 months. Each child returns for three more visits in the first year, two in the second and then once annually until adulthood.

Since recruitment began in 1985, detailed data on diet, growth, development, and metabolism between infancy and adulthood have been collected from over 1,300 healthy children. However, the children who were initially recruited for the DONALD Study differed considerably in age and prospectively collected data on breastfeeding was not always available. In addition, because of the open cohort design, many DONALD participants had not yet reached young adulthood by the time of this analysis. Finally, adult participants are invited to provide a fasting blood sample only since 2004. However, when this change to the study's design became effective, many participants did not accept our invitation to continue on the extension of the DONALD Study into adulthood. Therefore,

- 592 term (37 – 42 week gestation) singletons with a birth weight >2,500 g had returned at the age of 18 years or older and provided at least one anthropometric measurement in young adulthood. Among these,
- a fasting blood sample had been collected between 2004 and 2012 in 335 participants. Of these,
- 276 had also provided prospectively collected data on breastfeeding during infancy. Finally,
- data on all relevant confounders, e.g. maternal overweight, parental education, and early life characteristics, was available for 233 participants (125 women, 108 men; age range 18–37 years, mean age = 22.4 years).

This sample of 233 participants was used for analyses on IGF-I and IGFBP-3. Values of IGFBP-1 and IGFBP-2 were available in 229 participants, HOMA-IR could be calculated for 232 participants.

### Breastfeeding

At the initial visit (i.e. age 3 or 6 months) the study pediatrician questioned mothers about how long (in weeks) they had fully breastfed their infant (no solid foods and no liquids other than breast milk, tea or water). If the mother was still fully breastfeeding, this question was repeated at each subsequent visit(s) (e.g. 6, 9, or 12 months) until complementary feeding was initiated. In addition, for >70% of the infants, their mothers had kept weighed 3-day dietary records during the first year of life so that infant feeding could be quantified at 3, 6, 9, or 12 months. The study dietitian also questioned these mothers about when they had first started feeding formula or solid foods. Consistency checks comparing data collected by the pediatricians, the recording of breast milk, and information acquired by the dietitians were performed so as to eliminate any potential source of error.

In the present analysis, infants who were not fully breastfed for up to 2 weeks were classified as “never fully breastfed”. The remaining “ever fully breastfed” ones were further divided into those breastfed for a “short” duration (i.e. fully breastfed for more than 2 weeks up to a maximum of 17 weeks), and those breastfed for a “long” duration (full breastfeeding for more than 17 weeks (e.g. >4 months)) [24].

### Blood data

Venous blood samples were drawn after an overnight fast, immediately centrifuged, and stored <4°C for subsequent serum measurements of glucose. Glucose was routinely determined using an automated analyzer (ADVIA 1650, Siemens Healthcare Diagnostics). Blood samples were frozen at -80°C and then shipped to the Laboratory for Translational Hormone Analytics in Paediatric Endocrinology at the University of Giessen where they were analyzed for IGF-I and IGFBP-3 using a Radioimmunoassay (RIA, according to [25]), for IGFBP-2 and IGFBP-1 with an enzyme immunoassay (ELISA, Mediagnost, Germany; lot 061010 and lot 050910, respectively), as well as for plasma insulin concentrations using an immunoradiometric assay (IRMA, DRG Diagnostics, Germany; lot 120904). HOMA-IR was used as a marker of insulin resistance and calculated using the following formula: fasting insulin [mU mL^−1^] x fasting glucose [mmol L^−1^]/22.5) [26].

### Anthropometric data and calculations

Participants were measured at each visit according to standard procedures [27], dressed in underwear only and barefoot. From the age of 2 years onward, standing height was measured to the nearest 0.1 cm using a digital stadiometer (Harpenden Ltd., Crymych, UK). Body weight was measured to the nearest 100 g using an electronic scale (Seca 753E; Seca Weighing and Measuring Systems, Hamburg, Germany). Waist circumference in younger adulthood was measured at the midpoint between the lower rip and the iliac crest to the nearest 0.1 cm.

Skinfold thickness was measured from the age of 6 months onward at four different sites (suprailiacal, subscapular, biceps, triceps) on the right side of the body to the nearest 0.1 mm using a Holtain caliper (Holtain Ltd., Crosswell, United Kingdom). The three trained nurses who performed the measurements undergo an annual quality control, conducted with 6 to 8 healthy young adult volunteers. Average inter- and intra-individual variation coefficients obtained in the last eight years (2005 – 2012) were 9.1 and 12.1% for biceps, 4.7 and 5.8% for triceps, 4.3 and 7.4% for subscapular, and 7.9 and 9.0% for supra-iliacal skinfolds, which indicates moderate reliability and is comparable to results of large-scale epidemiologic studies in adults [28]–[30].

Body fat percentage (BF%) in adults was estimated from skinfolds using Durnin and Womersley equations, which are based on triceps, biceps, scapular and iliacal skinfolds [31], and used to obtain FM and FFM. FMI and FFMI (in kg/m^2^) during puberty and adulthood was calculated using the following formula: FMI =  weight x BF% / height^2^ and FFMI =  [weight – (weight x BF%) / height^2^)]. We chose to investigate FMI rather than BF% as the use of this measure has recently been criticized to incorrectly reflect body-size-adjusted adiposity [32].

### Early life and socioeconomic characteristics

On their child's admission to the study, parents were interviewed by the study pediatrician, and weighed and measured by the study nurses using the same equipment as for children from 2 years onward. Information on the child's birth characteristics was abstracted from the ‘Mutterpass’, a standardized document given to all pregnant women in Germany.

### Statistical analysis

Descriptive characteristics of the study sample are presented as frequencies or medians (P25; P75) according to duration of full breastfeeding (i.e. never, short, long). Tests for differences across breastfeeding groups were performed using the Chi-Square-Test for categorical and the Kruskal Wallis-Test for continuous variables.

We used two different statistical approaches to investigate the association of breastfeeding with FMI, FFMI, WC, the GH-IGF-axis and HOMA-IR. Firstly, multiple linear regression models were applied to evaluate whether a linear trend across groups of breastfeeding duration (never, short, long) existed on mean outcome levels, assigning each category its median breastfeeding duration (0, 13 and 25 weeks, respectively) and modelling this variable continuously. Since apart from IGF-I and IGFBP-3 the outcomes were not normally distributed, log-transformation was performed and values were transformed back for the ease of interpretability. Secondly, quantile regression was applied. Quantile regression enables to model different sample percentiles (“quantiles”) of an outcome variable with respect to covariates [20]. The approach and interpretation of quantile regression are similar to those of linear regression, but it leads to more comprehensive results due to its ability to assess the relevance of an exposure for any part of the outcome distribution, while linear regression can model only the effect on the mean of the outcome. For the present analyses, we assessed the 10^th^, 25^th^, 50^th^, 75^th^ and 90^th^ percentiles for all outcomes.

Potential confounders were evaluated in separate linear regression models and included either based on an *a priori* decision or if they modified the regression coefficient for breastfeeding by >10% [33]. These covariates were then also adopted for the quantile regression. Variables tested in this way included age in adulthood (years), maternal overweight (BMI ≥25 kg/m^2^ yes/no), high maternal and paternal education (12 years schooling yes/no), paternal university degree (yes/no), maternal age at birth, smoking in the household (yes/no), gestational age (37–38/39–40/41–42 weeks), pregnancy weight gain (>15 kg yes/no), parity (firstborn yes/no), birth weight (<3000 g/3000–<3500 g/≥3500 g), and whether birth weight and length were appropriate-for-gestational age (i.e. between the 10th and 90th percentiles of the German sex-specific birth weight and height-for-gestational age curves yes/no [34]). Since breastfeeding might affect later insulin metabolism by increasing (central) body fat we added adult waist circumference to the final models with HOMA-IR and IGFBP-2 as outcomes in an additional step, in order to evaluate potential mediation by obesity. Interactions between breastfeeding and sex were tested but did not reach significance in the basic or final models (p>0.1). Stratified analyses, however, indicated considerable sex differences. Therefore, all analyses were performed separately for women and men.

The statistical analyses were carried out using SAS procedures (version 9.3, SAS Institute, Cary, NC, USA) and the open-source software R 2.14.2 (http://cran.r-project.org), using the quantreg package. A p-value <0.05 (two-sided) was considered statistically significant.

## Results

About two thirds of both women and men had ever been fully breastfed in infancy (68.8 and 64.8% respectively). Median breastfeeding duration in those ever fully breastfed was 17 weeks (range 3–43 weeks). General characteristics of the study sample according to breastfeeding duration are presented in **Table 1**. Among women who had not been breastfed, more were overweight in young adulthood and more had an overweight mother than among those breastfed for a short or long duration.

**Table 1:** Characteristics of the study sample according to duration of full breastfeeding in infancy, DONALD Study (n = 125 women, 108 men)^a^.

	Women				Men			
	Duration of full breastfeeding				Duration of full breastfeeding			
Variable	Never (≤2 weeks)	Short (3–17 weeks)	Long (>17 weeks)	p^b^	Never (≤2 weeks)	Short (3–17 weeks)	Long (>17 weeks)	p^b^
**n (%)**	39 (31.2)	48 (38.4)	38 (30.4)	-	38 (35.2)	44 (40.7)	26 (24.1)	-
**Early life**								
Birth weight (kg)	3.5 (3.2; 3.8)	3.3 (3.0; 3.7)	3.5 (3.1; 3.8)	0.3	3.4 (3.3; 3.8)	3.6 (3.3; 4.0)	3.5 (3.1; 3.8)	0.3
n (%) Birth weight ≥3,500 g	18 (46.2)	18 (37.5)	19 (50.0)	0.5	16 (42.1)	26 (59.1)	13 (50.0)	0.3
Birth length (cm)	51 (50; 53)	51 (50; 52)	52 (50; 53)	0.3	52 (50; 53)	53 (51; 54)	52 (51; 53)	0.1
n (%) AGA^c^	24 (61.5)	40 (83.3)	30 (79.0)	0.05	29 (76.3)	36 (81.8)	21 (80.8)	0.8
n (%) Gestational age 39–40 weeks	23 (59.0)	34 (70.8)	24 (63.2)	0.6	25 (65.8)	25 (56.8)	15 (57.7)	0.9
n (%) Pregnancy weight gain >15 kg	8 (20.5)	12 (25.0)	8 (21.1)	0.9	5 (13.2)	10 (22.7)	7 (26.9)	0.4
n (%) Firstborn	29 (74.4)	30 (62.5)	21 (56.8)	0.3	23 (60.5)	28 (63.6)	12 (46.2)	0.3
Maternal age at birth (years)	29 (27; 34)	29 (27; 32)	30 (28; 33)	0.6	29 (26; 32)	30 (28; 32)	30 (28; 35)	0.2
**Young adulthood**								
Age (years)	24.1 (18.2; 26.8)	21.6 (18.1; 24.7)	21.6 (18.3; 24.0)	0.3	23.2 (21.1; 28.1)	21.2 (18.1; 23.0)	21.7 (18.0; 22.7)	0.01
n (%) Overweight	14 (35.9)	6 (12.5)	4 (10.5)	0.01	16 (42.1)	10 (22.7)	8 (30.8)	0.2
**Family characteristics**								
n (%) Smoking in the household	16 (41.0)	17 (35.4)	10 (26.3)	0.4	15 (39.5)	17 (38.6)	11 (42.3)	0.95
n (%) Maternal overweight	15 (38.5)	15 (31.3)	5 (13.2)	0.04	15 (39.5)	11 (25.0)	6 (23.1)	0.3
n (%) Mother ≥12 years schooling	14 (35.9)	25 (52.2)	20 (52.6)	0.2	14 (36.8)	22 (50.0)	14 (53.9)	0.3
n (%) Father University degree	15 (38.5)	27 (56.3)	22 (57.9)	0.2	15 (39.5)	19 (43.2)	13 (50.0)	0.7

a Data are frequencies (%) or medians (25^th^ percentile; 75^th^ percentile). Abbreviations used: DONALD, Dortmund Nutritional and Anthropometric Longitudinally Designed; AGA, appropriate-for-gestational age. Missing values: n = 1 for firstborn status.

b Test for differences between breastfeeding groups based on the Chisquare-test for categorical and the Kruskal Wallis-Test for continuous variables.

c According to [34].

### Adult GH-IGF-axis / insulin sensitivity

In neither women nor men, breastfeeding duration was associated with mean adult concentrations of IGF-I, IGFBP-1 or IGFBP-3 (**Table 2**). In men, there was no linear relation of breastfeeding duration with either IGFBP-2 or HOMA-IR either. By contrast, women who had been breastfed for a longer duration had higher concentrations of IFGBP-2 (difference between long and no breastfeeding +51 µg L^−1^, p_trend_ over breastfeeding categories = 0.02) and lower values of HOMA-IR (difference -0.6 units, p_trend_ = 0.003). Additional consideration of WC as a potential mediator of these associations attenuated the relation for IGFBP-2 towards non-significance (adjusted means (95% CI) no breastfeeding: 133 (106; 168), short breastfeeding: 137 (108; 173), long breastfeeding: 164 (126; 214) µg L^−1^; p_trend_ = 0.15), but not that for HOMA-IR (no breastfeeding: 2.7 (2.4; 3.0) vs. short: 2.6 (2.4; 2.9) vs. long breastfeeding: 2.2 (2.0; 2.5); p_trend_ = 0.02, data not shown in Tables).

**Table 2:** Components of the GH-IGF-axis and insulin sensitivity in young adulthood according to breastfeeding status in infancy, DONALD Study (n = 122–125 women, 107–108 men)^a^.

	Duration of full breastfeeding			
Outcome	Never (≤2 weeks)	Short (3–17 weeks)	Long (>17 weeks)	p_trend_
***Women***				
**IGF-I (ng ml^−1^)**				
Model 1^b^	256 (226; 286)	256 (230; 283)	227 (196; 257)	0.2
Model 2^c^	248 (212; 285)	244 (207; 281)	222 (182; 262)	0.2
**IGFBP-1 (µg L^−1^)**				
Model 1	10.4 (7.7; 14.2)	9.5 (7.2; 12.5)	10.8 (7.9; 14.9)	0.9
Model 2	8.2 (5.5; 12.2)	6.9 (4.6; 10.4)	8.2 (5.3; 12.8)	0.99
**IGFBP-3 (mg L^−1^)**				
Model 1	3.7 (3.5; 4.0)	3.7 (3.4; 3.9)	3.6 (3.3; 3.9)	0.4
Model 2	3.5 (3.2; 3.9)	3.5 (3.2; 3.9)	3.4 (3.1; 3.8)	0.7
**IGFBP-2 (µg L^−1^)**				
Model 1	121 (100; 146)	127 (107; 151)	163 (134; 198)	0.04
Model 2	127 (100; 160)	142 (112; 181)	178 (136; 233)	0.02
**HOMA-IR**				
Model 1	2.7 (2.4; 3.0)	2.7 (2.4; 2.9)	2.2 (2.0; 2.5)	0.01
Model 2	2.8 (2.5; 3.1)	2.6 (2.4; 2.9)	2.2 (1.9; 2.4)	0.004
***Men***				
**IGF-I (ng ml^−1^)**				
Model 1	243 (215; 271)	252 (226; 277)	261 (228; 293)	0.4
Model 2	247 (215; 280)	255 (224; 286)	265 (228; 303)	0.4
**IGFBP-1 (µg L^−1^)**				
Model 1	5.5 (3.9; 7.9)	5.6 (4.1; 7.8)	4.7 (3.1; 7.1)	0.6
Model 2	5.5 (3.7; 8.3)	5.5 (3.7; 8.2)	4.8 (3.0; 7.7)	0.6
**IGFBP-3 (mg L^−1^)**				
Model 1	3.3 (3.0; 3,6)	3.5 (3.2; 3.7)	3.2 (2.8; 3.5)	0.9
Model 2	3.1 (2.8; 3.5)	3.3 (3.0; 3.7)	3.1 (2.7; 3.5)	0.9
**IGFBP-2 (µg L^−1^)**				
Model 1	173 (145; 206)	209 (178; 246)	181 (147; 224)	0.6
Model 2	167 (131; 213)	204 (162; 257)	179 (137; 234)	0.6
**HOMA-IR**				
Model 1	2.5 (2.1; 2.9)	2.4 (2.1; 2.7)	2.8 (2.3; 3.3)	0.4
Model 2	2.4 (2.1; 2.8)	2.3 (2.0; 2.7)	2.8 (2.3; 3.3)	0.3

a Data are adjusted means (95% CI). Abbreviations used: DONALD, Dortmund Nutritional and Anthropometric Longitudinally Designed; GH, growth hormone; IGF-I: insulin-like-growth-factor-1; IGFBP: insulin-like growth factor binding protein; HOMA: homoeostatic model assessment; IR: insulin resistance. Missing values: n = 1 for firstborn status.

b Model 1: adjusted for age in adulthood.

c Model 2 for IGF-I, IGBP-1, IGFBP-3: adjusted for age in adulthood, paternal schooling ≥12 years (yes/no), firstborn status (yes/no), birth weight and length (appropriate for gestational age yes/no), gestational age (37–38/39–40/41–42 weeks), smoking in the household (yes/no). Model 2 for IGFBP-2: adjusted for age in adulthood, maternal overweight (yes/no), paternal schooling ≥12 years (yes/no), birth weight (<3000g/3000–<3500 g/≥3500 g), gestational age (37–38/39–40/41–42 weeks). Model 2 for HOMA-IR: for age in adulthood, maternal overweight (yes/no), paternal schooling ≥12 years (yes/no), firstborn status (yes/no).

**Figure 1:**
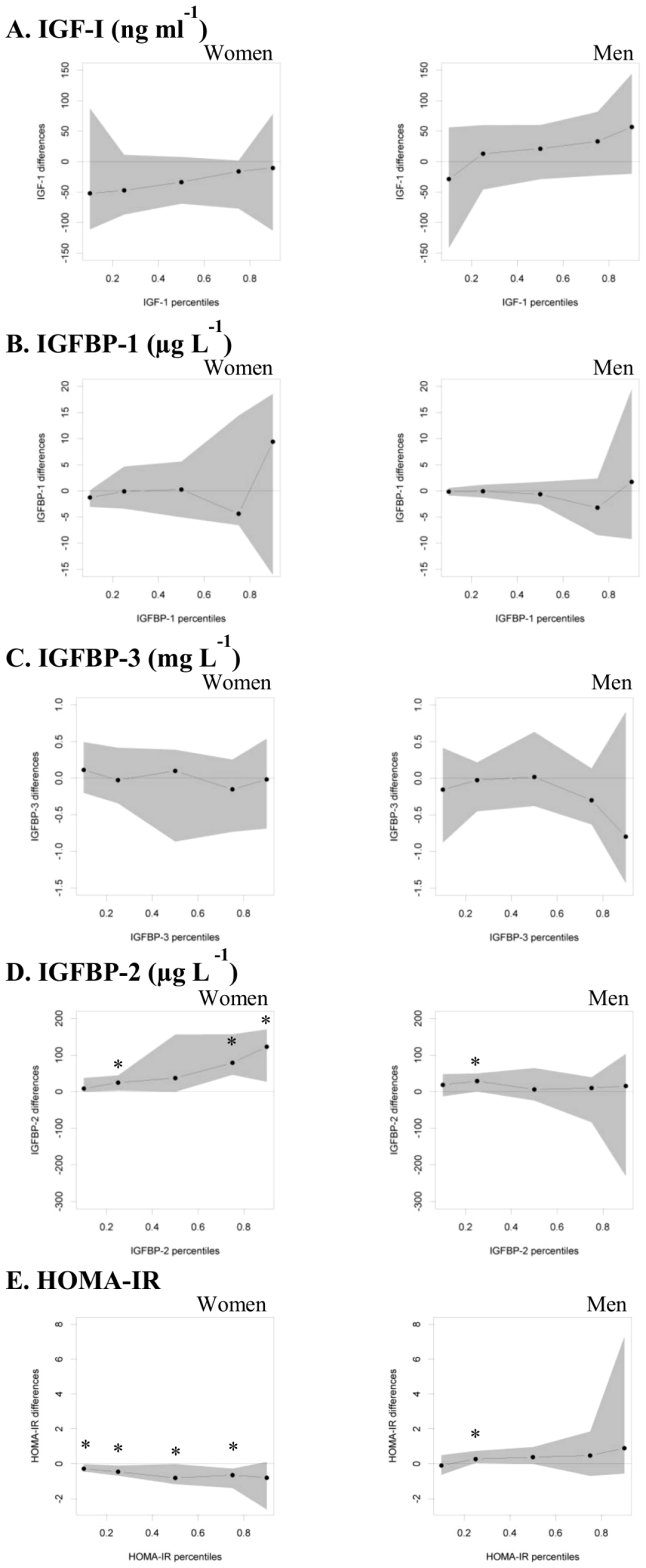
GH-IGF-axis and HOMA-IR in young adulthood according to breastfeeding duration in multivariable quantile regression models. Displayed are point estimates (95% CI) for IGF-I (A), IGFBP-1 (B), IGFBP-3 (C), IGFBP-2 (D) and HOMA-IR (E) differences between women and men breastfed for a long duration (i.e. >17 weeks) vs. those not breastfed (i.e. ≤2 weeks) for specific percentiles (10^th^, 25^th^, 50^th^, 75^th^ and 90^th^ percentiles). Models included age in adulthood, maternal overweight (yes/no), paternal university degree (yes/no), firstborn status (yes/no), smoking in the household (yes/no) in the case of FMI and WC; in the case of FFMI: age in adulthood, maternal overweight (yes/no), paternal university degree (yes/no), birth weight and length (appropriate for gestational age yes/no), firstborn status (yes/no), smoking in the household (yes/no). DONALD Study, n = 228-232. * p<0.05 DONALD, Dortmund Nutritional and Anthropometric Longitudinally Designed; IGF-I, insulin-like growth factor 1, IGFBP, insulin-like growth factor binding protein, HOMA-IR, homeostasis model assessment for insulin resistance.

**Figure 1** summarizes the results of the quantile regression models. As in the linear regression, long breastfeeding was not associated with IGF-I, IGFBP-1 or IGFBP-3 in both sexes. In men, long breastfeeding was significantly related to higher concentrations of IGFBP-2 and lower HOMA-IR values at the 25^th^ percentile only. In women, the association of breastfeeding with IGFBP-2 was stronger at higher percentiles: in those breastfed for a long duration, IGFBP-2 was 24.8 (95% CI: 2.9; 44.3) µg L^−1^ higher at the 25^th^, 78.9 (45.9; 156.7) µg L^−1^ higher at the75^th^, and 122.6 (26.9; 170.2) µg L^−1^ higher at the 90^th^ percentile when compared to those who were not breastfed in infancy. Their HOMA-IR values were lower at the 10^th^, 25^th^, 50^th^, and 75^th^ percentiles (difference to those not breastfed ranging from -0.3 to -0.8 units, all p<0.05).

### Adult body composition / body fat distribution

Breastfeeding was not related to mean adult FMI, FFMI or WC in men (**Table 3**). In women, prolonged breastfeeding was associated with a lower mean FMI, FFMI, and WC in young adulthood in the basic model (Model 1) as well as after consideration of early life and family characteristics (Model 2, p for a linear trend across breastfeeding categories = 0.004–0.02).

**Table 3:** Body composition and body fat distribution in young adulthood according to breastfeeding duration in infancy, DONALD Study (n = 125 women, 108 men)^a^.

	Duration of full breastfeeding			
Outcome	Never (≤2 weeks)	Short (3–17 weeks)	Long (>17 weeks)	p_trend_
***Women***				
**Fat mass index (kg/m^2^)**				
Model 1^b^	7.9 (7.2; 8.8)	6.8 (6.2; 7.4)	6.1 (5.5; 6.8)	0.001
Model 2^c^	7.9 (7.1; 8.7)	6.8 (6.2; 7.5)	6.4 (5.7; 7.2)	0.01
**Fat-free mass index (kg/m^2^)**				
Model 1	16.2 (15.7; 16.7)	15.2 (14.8; 15.6)	15.1 (14.6; 15.6)	0.003
Model 2	16.1 (15.6; 16.6)	15.1 (14.6; 15.6)	15.2 (14.6; 15.8)	0.02
**Waist circumference (cm)**				
Model 1	76.2 (73.8; 78.7)	72.6 (70.5; 74.7)	70.2 (67.9; 72.5)	0.001
Model 2	75.8 (73.2; 78.5)	72.4 (70.2; 74.7)	70.5 (67.9; 73.2)	0.004
***Men***				
**Fat mass index (kg/m^2^)**				
Model 1	4.5 (3.9; 5.3)	3.9 (3.4; 4.5)	4.3 (3.6; 5.2)	0.6
Model 2	4.5 (3.8; 5.2)	3.8 (3.3; 4.4)	4.3 (3.6; 5.2)	0.6
**Fat-free mass index (kg/m^2^)**				
Model 1	19.3 (18.7; 19.9)	18.8 (18.3; 19.4)	19.6 (18.9; 20.3)	0.7
Model 2	19.3 (18.7; 19.9)	18.9 (18.3; 19.5)	19.6 (18.9; 20.4)	0.7
**Waist circumference (cm)**				
Model 1	84.4 (81.7; 87.1)	79.8 (77.5; 82.1)	83.3 (80.2; 86.6)	0.5
Model 2	84.2 (81.4; 86.9)	79.5 (77.0; 82.0)	83.3 (80.1; 86.6)	0.5

a Data are adjusted means (95% CI). Abbreviations used: DONALD, Dortmund Nutritional and Anthropometric Longitudinally Designed. Missing values: n = 1 for firstborn status.

b Model 1: adjusted for age in adulthood.

c Model 2 for FMI, WC: adjusted for age in adulthood, maternal overweight (yes/no), paternal university degree (yes/no), firstborn status (yes/no), smoking in the household (yes/no). Model 2 for FFMI: adjusted for age in adulthood, maternal overweight (yes/no), paternal university degree (yes/no), birth weight and length (appropriate for gestational age yes/no), firstborn status (yes/no), smoking in the household (yes/no).

**Figure 2:**
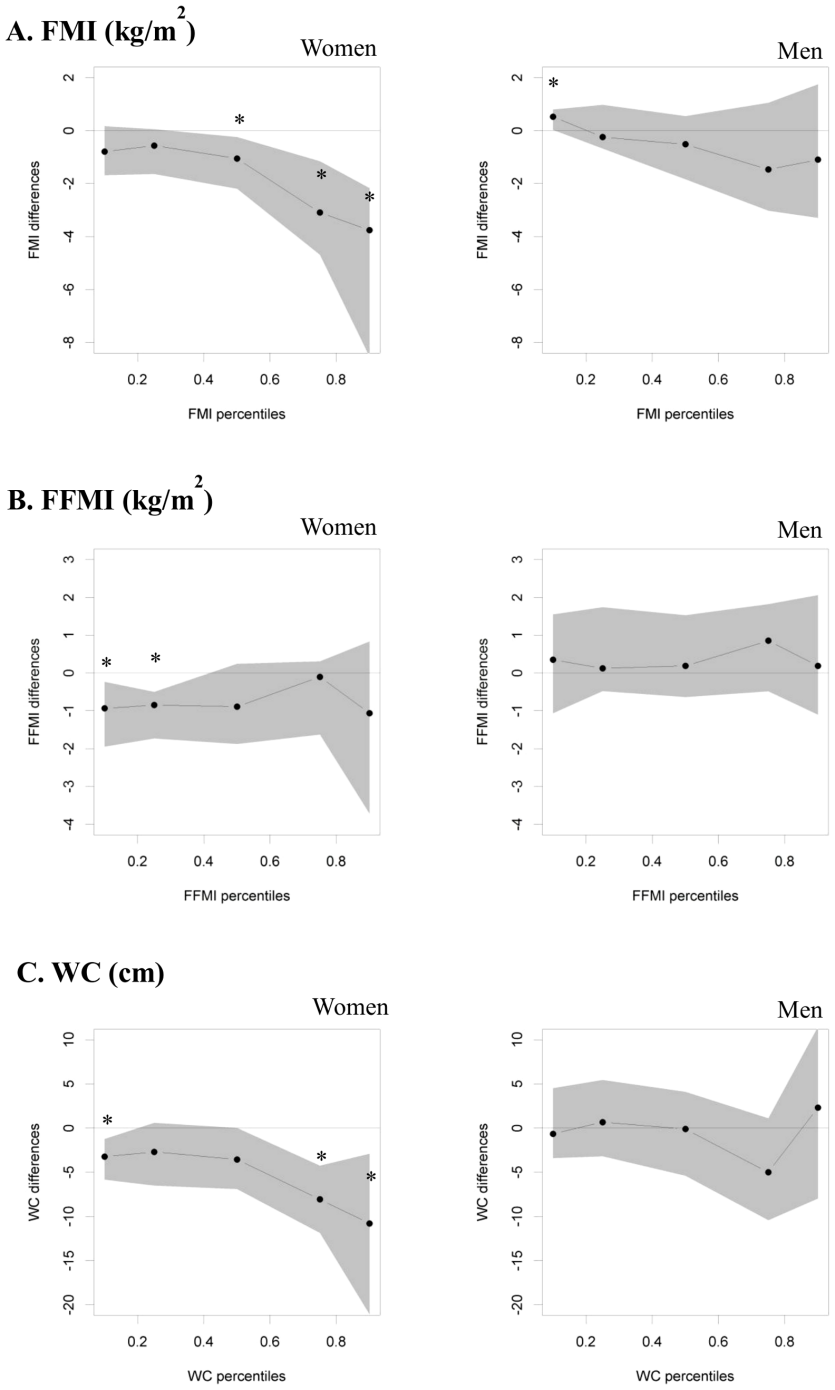
Body composition measures in young adulthood according to breastfeeding duration in multivariable quantile regression models. Displayed are point estimates (95% CI) for FMI (A), FFMI (B) and WC (C) differences between women and men breastfed for a long duration (i.e. >17 weeks) vs. those not breastfed (i.e. ≤2 weeks) for specific percentiles (10^th^, 25^th^, 50^th^, 75^th^ and 90^th^ percentiles) in multivariable quantile regression models. Models included age in adulthood, maternal overweight (yes/no), paternal university degree (yes/no), firstborn status (yes/no), smoking in the household (yes/no) in the case of FMI and WC, and age in adulthood, maternal overweight (yes/no), paternal university degree (yes/no), birth weight and length (appropriate for gestational age yes/no), firstborn status (yes/no), smoking in the household (yes/no) in the case of FFMI. DONALD Study, n = 232. * p<0.05 DONALD, Dortmund Nutritional and Anthropometric Longitudinally Designed; FFMI, fat-free mass index; FMI, fat mass index; WC, waist circumference.

The results of the quantile regression showed that the effect of longer full breastfeeding vs. no breastfeeding on women's FMI was particularly pronounced at higher percentiles (**Figure 2**): In those breastfed for a long duration, FMI in young adulthood was −1.1 kg/m^2^ (95% CI: −2.2; −0.2 kg/m^2^) lower at the 50^th^ percentile, −3.1 (−4.7; −1.2) kg/m^2^ lower at the 75^th^ percentile, and −3.8 (−8.5; −2.2) kg/m^2^ lower at the 90^th^ percentile. No significant association existed at the 10^th^ or 25^th^ percentile. Reductions in WC also increased across the percentiles (from −3.2 at the 10^th^ to −10.8 cm at the 90^th^ percentile), but were significant at both the lower and upper end of the distribution, i.e. the 10^th^, 75^th^, and 90^th^ percentile. Point estimates for the association of long breastfeeding with adult FFMI were more similar across the FFMI distribution, but statistically significant lower values were confined to the 10^th^ and 25^th^ percentiles.

No such patterns were observed in men with respect to FFMI and WC. Comparable to women however point estimates for FMI differences tended to become larger in men at the upper percentiles (i.e., a 1.5 and 1.1 kg/m^2^ lower FMI in those breastfed for a long duration at the 75^th^ and 90^th^ percentile, respectively, as compared to those who had not been breastfed), but the only significant association existed at the 10^th^ percentile. Here, males breastfed for a long duration had a 0.5 (0.0; 0.8) kg/m^2^ higher FMI.

## Discussion

The results of our study suggest that breastfeeding has long lasting beneficial effects on adiposity measures as well as insulin sensitivity among women, as reflected by higher mean values of IGFBP-2 and lower values of HOMA-IR. Except for HOMA-IR, these relations were particularly pronounced at the upper tails of the outcome distribution. However, we did not see similar associations in men. Furthermore, our findings argue against a programming effect of breastfeeding on the adult GH-IGF-axis in both sexes.

Breastfed infants are characterized by lower values of IGF-I in early and late infancy [13], [15], [35]–[37], while previous studies reported either no [36], [13], [18] or inverse associations [35], [38] for IGFBP-3 in infancy and childhood. The inverse association between breastfeeding and IGF-I levels over the short-term may, however, reverse over the long-term, at least when looking at certain periods (e.g. the end of the growth years): A Danish cohort found higher IGF-I in breastfed children at the age of 17 years, albeit non-significant [37]. Pituitary resetting in response to the reduced ambient IGF-I concentrations in breastfed children has been suggested, i.e. lowered thresholds for stimulating GH release and thus increased IGF-I output later on [39], [17]. Additional support for such a phenomenon comes from the inverse association of IGF-I at 9 months with concentrations at 17 years seen in a Danish study [37], as well as higher IGF-I found in British children who had been breastfed as infants [18]. In addition, intake of cow's milk also seems to exert opposing acute and long-term effects [39]. These findings indeed suggest that the GH-IGF-axis can be programmed via the mechanisms outlined above. Our study, the first to investigate the association of breastfeeding and IGF-I beyond adolescence, does not confirm the resetting hypothesis with respect to breastfeeding. Also, we found no association with IGFBP-1, IGFBP-3 or the ratio of IGF-I/IGFBP-3 (perhaps reflecting free IGF-I, data not shown). Interestingly, however, a previous analysis of the DONALD cohort suggested that habitually higher animal protein intakes in early life may exert a long-term programming of the GH-IGF-axis in males and indicated a reversal in this association between early life and adolescence [40].

Our study adds epidemiologic evidence that breastfeeding favorably affects body fatness and central adiposity beyond childhood, as evident from the fact that women breastfed for a longer duration had a 19% lower mean FMI, accompanied by a smaller WC, but only a 6% lower FFMI as compared to those never breastfed. A previous meta-analysis suggested that breastfeeding primarily affects later overweight risk, i.e. no association of breastfeeding on mean continuous BMI in childhood was found [41]. While we observed benefits also for mean adiposity outcome levels, the quantile regression approach indeed revealed that these results were driven by a shift in the upper tail only. For WC, there was an inverse association at the lowest percentile too, but comparable to FMI, point estimates became larger at the higher percentiles. Since we did not observe regression-to-the mean effects, results were still significant for the overall mean in the linear models. Beyerlein et al. [20], by contrast, found such differential associations with breastfeeding yielding higher childhood BMI levels at the lower end and higher levels at the upper end of the BMI distribution.

We also observed lower HOMA-IR values as well as higher concentrations of IGFBP-2 among women breastfed for a longer duration. Both findings point towards higher insulin sensitivity [11], in the case of IGFBP-2 also towards a reduced cancer risk [42]. Higher IGFBP-2 concentrations in breastfed infants have been observed at 6 months already [38]. It is also known that formula feeding stimulates insulin secretion more than breastfeeding, presumably due to its higher protein content [12]. Our results indicate that a long-term set point change and accordingly development of insulin resistance could be the consequence. However, evidence from other studies is controversial. While two studies found an association between breastfeeding and markers of insulin resistance in overweight and obese children [43] and adults [44], others did not see an association between breastfeeding and insulin sensitivity at ages 9–15 [45] or 45–59 years [46]. Furthermore, conclusions may be hampered by methodological shortcomings, since previous studies have in common that they assessed breastfeeding retrospectively and/or lacked information on breastfeeding exclusivity and duration. It is also very likely that some of the effect of breastfeeding on insulin metabolism is secondary to effects on body composition. In accordance with this, in our study additional consideration of WC attenuated the association with IGFBP-2 towards non-significance, however not that with HOMA-IR.

Our findings of a beneficial effect of breastfeeding were largely confined to women, with respect to both anthropometry and insulin metabolism. In the quantile regression, single significant differences between long and no breastfeeding were also found in men, but are difficult to interpret and may have been the result of by-chance associations. It is well described that in early life girls have higher values of IGF-I and IGFBP-3 than boys, who in turn show higher IGF-I levels in late puberty [17]. In addition, girls have been proposed to be intrinsically more insulin resistant than boys and could be more susceptible to breastfeeding in their hormonal responses and growth [47]. In a randomized controlled trial of infants fed formula with either low or high protein content, influences on IGF-I and IGFBP-2 levels were more pronounced for girls. However, in that study no differences in insulin secretion (reflected by urinary C-peptide) or anthropometry were seen in the first months [48]. Since previous studies on breastfeeding and adult health outcomes did not differentiate between women and men, future studies should specifically investigate whether infant feeding induces sex-specific programming pathways via initial hormonal responses and/or growth patterns.

The clear strength of the current analysis lies in the carefully collected, prospective data on breastfeeding duration, and in our ability to consider important potential confounders such as parental and early life characteristics. It thus fulfills several crucial methodological pre-requisites for a study to contribute to the elucidation of long-term health effects of breastfeeding [49]. Also, the innovative procedure of quantile regression allowed a more comprehensive analysis of the data, in comparison to standard linear or logistic regression [20].

Still, our study has several limitations. Firstly, it is purely observational and hence, any conclusions have to be drawn with caution. We cannot exclude that our findings are biased by residual confounding, i.e. that they are actually due to differences in socio-economic and lifestyle factors that have not been eliminated statistically, instead of a true metabolic effect of breastfeeding. Secondly, we determined FMI and FFMI on the basis of skinfold thickness measurements, which have a higher susceptibility to measurement error than specialized research methods such as hydrodensitometry [50] or magnetic resonance imaging [51]. Yet, the skinfold equations of Durnin and Womersley agree, on average, very well with results from hydrodensitometry [50]. Thirdly, our analysis is based on single blood measurements in younger adulthood to represent long-term circulating levels of the GH-IGF-axis and HOMA-IR as a measure of insulin resistance. However, IGF-I values were reported to have a low intra-individual variation [52] and HOMA-IR is considered a reasonable method to assess peripheral insulin sensitivity in epidemiological studies [53]. Fourthly, the participants of the DONALD Study are characterized by a high socio-economic status as compared to the German population, which may however have reduced our vulnerability to residual confounding. It is further worthwhile mentioning that a nationwide German study on breastfeeding conducted 1997-1998 found comparable numbers with respect to prevalence and duration of breastfeeding [22].

In conclusion, our study suggests that breastfeeding has long-term, favorable effects on adult body composition, body fat distribution and insulin metabolism in women, but not in men. In both sexes, the results argue against a programming effect of infant nutrition on the GH-IGF-axis.
